# Endophthalmitis following intravitreal injection of anti-VEGF agents: long-term outcomes and the identification of unusual micro-organisms

**DOI:** 10.1186/s12348-015-0069-5

**Published:** 2016-01-12

**Authors:** Mira M. Sachdeva, Ala Moshiri, Henry A. Leder, Adrienne W. Scott

**Affiliations:** The Wilmer Eye Institute, Johns Hopkins Hospital, 1800 Orleans Street, Baltimore, MD USA; UC Davis Eye Center, Sacramento, CA USA; Elman Retina Group, 9114 Philadelphia Road, Suite 310, Baltimore, MD USA

**Keywords:** Endophthalmitis, Intravitreal injection, Vascular endothelial growth factor

## Abstract

**Background:**

While the development of targeted molecular therapy to inhibit vascular endothelial growth factor (VEGF) has revolutionized the treatment and visual prognosis of highly prevalent retinal diseases such as diabetic retinopathy and age-related macular degeneration, each intravitreal injection of these agents carries a small risk of endophthalmitis which can be visually devastating. In the absence of specific guidelines, current management of post-injection endophthalmitis is typically extrapolated from data regarding endophthalmitis occurring after cataract surgery despite potential differences in pathogenic organisms and clinical course. Here, we assess the contribution of intravitreal injections of anti-VEGF agents to all cases of endophthalmitis at our tertiary care referral center and characterize the clinical outcomes and microbial pathogens associated with post-injection endophthalmitis in order to inform management of this serious iatrogenic condition.

**Results:**

During the 7-year study period analyzed, 199 cases of endophthalmitis were identified using billing records. Of these, the most common etiology was post-surgical, accounting for 62 cases (31.2 %), with bleb-associated, endogenous, and corneal ulcer-related infections representing the next most frequent causes, comprising 15.6 % (31/199), 13.1 % (26/199), and 13.6 % (27/199) of all cases, respectively. Intravitreal injections of anti-VEGF agents represented 8.5 % of endophthalmitis (17/199 cases). Intraocular cultures yielded positive results in 75 % of post-injection cases, with the majority associated with coagulase-negative *Staphylococcus*. Consistent with prior literature, a case of *Strep viridans* displayed more rapid onset and progression. We also report the first association of *Enterobacter cloacae* and *Lactococcus garvieae* with post-injection endophthalmitis. While all but one patient were treated with initial vitreous tap and intravitreal injection of antibiotics, both patients with these rare organisms exhibited persistent vitritis requiring subsequent vitrectomy. Long-term outcomes of post-injection endophthalmitis indicated visual recovery to baseline levels, even with resumption of anti-VEGF agents following resolution of the acute infection.

**Conclusions:**

Acute endophthalmitis following intravitreal injections of anti-VEGF agents is an uncommon but potentially devastating complication which may be managed effectively with vitreous tap and injection of intravitreal antibiotics. However, persistent vitritis requiring subsequent vitrectomy should raise suspicion for unusual pathogens.

## Background

While the advent of molecular therapeutics targeting vascular endothelial growth factor (VEGF) has revolutionized the management and visual prognosis for patients with age-related macular degeneration, retinal vein occlusion, diabetic macular edema, and other diseases resulting in retinal vascular leakage or choroidal neovascularization, each injection of these agents carries a small associated risk of endophthalmitis. Retrospective studies have suggested that the incidence of post-injection endophthalmitis ranges from 0.02 to 1.6 %, with several recent large-scale meta-analyses placing the incidence rates closer to 0.049 to 0.056 % [1–5]. Although the risk is low, the visual consequences can be devastating. While coagulase-negative *Staphylococcus* species have been isolated in the majority of culture-positive cases, endophthalmitis due to *Streptococcus* species has been associated with poorer visual outcomes [6–8]. Moreover, prophylactic topical antibiotic use may promote the emergence of unusual pathogens resistant to standard antimicrobial therapies; indeed, cases of atypical microbes isolated from intraocular fluid in patients with post-injection endophthalmitis have been increasingly reported [9–11]. Regarding the management of post-injection endophthalmitis, in the absence of evidence-based guidelines, many ophthalmologists use the severity of visual acuity on presentation to inform their decision to treat initially with either “tap and inject” (withdrawal of a vitreous sample for microbiologic analysis and intravitreal injection of antibiotics) versus primary vitrectomy based on the results and recommendations of the Endophthalmitis Vitrectomy Study (EVS) [12]. However, the characteristics of endophthalmitis following cataract surgery as studied in the EVS may differ from endophthalmitis following intravitreal injection in microbial etiology and clinical course. For example, *Streptococcus* species have been more likely implicated post-injection than post-operatively, suggesting a higher risk of iatrogenic infection due to oral flora during office-based procedures compared with those performed under sterile operating room conditions [4, 8, 13, 14]. In fact, a recent large retrospective analysis from France, where standard practice involves use of a face mask when performing intravitreal injections, described a much lower incidence of post-injection endophthalmitis due to *Streptococcus* species (only 1 case out of 23 culture-positive cases) compared with most studies from the USA [5]. Differences have even been described in clinical features of acute endophthalmitis following various types of intraocular surgery, including pars plana vitrectomy [13, 15]. A recent literature review highlighted the lack of current data regarding the most appropriate initial management of post-injection endophthalmitis [16].

In order to analyze the experience at our tertiary care academic institution, we reviewed all cases of endophthalmitis during a 7-year period and then specifically investigated the microbial pathogens and clinical course of those cases which occurred following intravitreal injection of an anti-VEGF agent to identify patterns which may inform treatment, especially in those cases caused by organisms other than coagulase-negative *Staphylococcus*. We also compared the microbial etiology to cases occurring after cataract surgery.

## Methods

The study protocol for this retrospective review was approved by the Institutional Review Board (IRB) of Johns Hopkins Hospital. The billing records of all patients treated in the general or subspecialty clinics from January 1, 2007 through December 31, 2013 at the Wilmer Eye Institute of Johns Hopkins Hospital were queried for the following associated International Classification of Diseases, Ninth Revision (ICD-9) billing codes, 360.00 (purulent endophthalmitis, unspecified), 360.01 (acute endophthalmitis), 360.03 (chronic endophthalmitis), and 360.19 (other endophthalmitis). Redundant entries were eliminated and the electronic medical record of each unique case was reviewed to determine the etiology of the endophthalmitis, presenting symptoms, culture results, and treatment.

With the exception of two patients who initially presented to our tertiary care institution with endophthalmitis several weeks after receiving intravitreal injection of an anti-VEGF agent and subsequent intravitreal antibiotics elsewhere (patients #8 and #11), all injections of intravitreal anti-VEGF agents were performed at the Wilmer Eye Institute with documentation of the procedure available for review. These were performed by eight different ophthalmologists with the use and duration of post-injection topical antibiotics at the discretion of the individual treating physician. In all cases, a lid speculum and non-sterile exam gloves were used. All patients received topical 5 % Betadine prior to injection with a 30-gauge needle administered in the inferotemporal quadrant 3.5 or 4 mm posterior to the limbus for pseudophakic or phakic patients, respectively.

The diagnosis of endophthalmitis was made clinically, and initial management was determined by each individual evaluating ophthalmologist. In all cases of endophthalmitis following anti-VEGF injection, aqueous or vitreous fluid was sent for microbial culture and culture results were reviewed. Regarding presentation of data, quantitative variables are expressed as the mean ± standard deviation and qualitative variables are expressed as percentages.

## Results

**Fig. 1:**
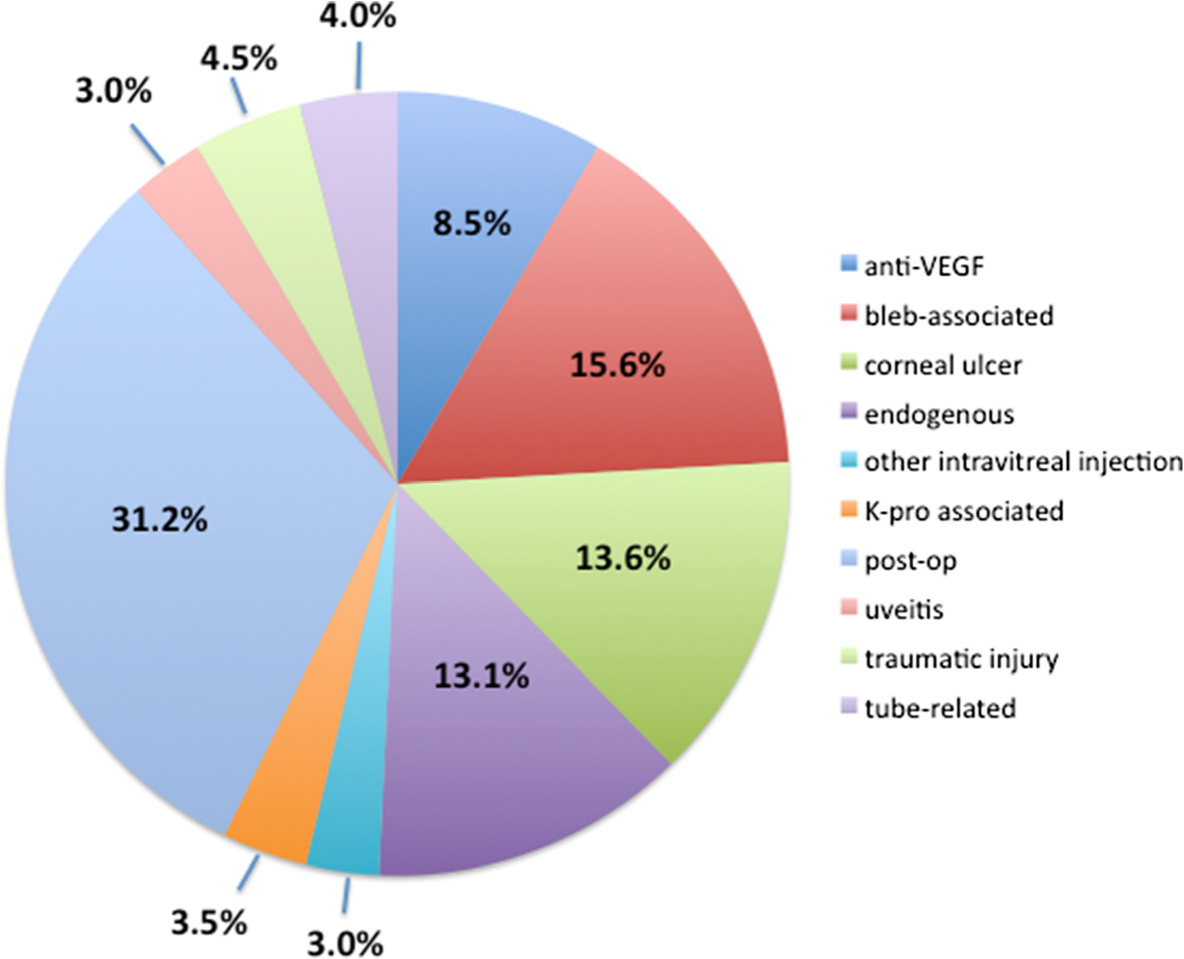
Etiology of all cases of endophthalmitis (2007 through 2013). The majority of cases occurred following intraocular surgery (post-op), which includes phacoemulsification (44 cases), Descemet’s stripping automated endothelial keratoplasty (DSAEK) (3 cases), penetrating keratoplasty (2 cases), and pars plana vitrectomy (PPV) (6 cases). Endophthalmitis following intravitreal injection of an anti-VEGF agent accounted for 8.5 % of the total number of cases

During the 7-year period queried, 267 patients were identified with the diagnosis of endophthalmitis based on billing records. Of these, 38 actually represented cases of asteroid hyalosis coded by the physician as 360.19 (other endophthalmitis) and thus were excluded from further analysis. Additionally, systematic chart review revealed no definitive etiology for 30 of the cases. Of the remaining 199 individual cases of endophthalmitis, 62 (31.2 %) occurred following intraocular surgery, including phacoemulsification (44 cases), Descemet’s stripping automated endothelial keratoplasty (DSAEK) (3 cases), penetrating keratoplasty (2 cases), and pars plana vitrectomy (PPV) (6 cases). Bleb-associated, endogenous, and corneal ulcer-related infections represented the next most frequent causes of endophthalmitis, comprising 15.6 % (31/199), 13.1 % (26/199), and 13.6 % (27/199) of all cases, respectively. The etiology of all cases is diagrammed in Fig. 1.

Intravitreal injections of anti-VEGF agents accounted for 8.5 % of all endophthalmitis cases (17/199 cases). Details were available for 16 of these 17 cases and are presented in Table 1. The indications for treatment included neovascular age-related macular degeneration, diabetic macular edema, macular edema related to vein occlusion, radiation retinopathy, and choroidal neovascularization secondary to multifocal choroiditis. In 10 patients, the drug administered was bevacizumab (Avastin; Genentech, Inc., South San Francisco, California, USA), and ranibizumab (Lucentis; Genentech, Inc., South San Francisco, California, USA) was used in 6 cases. The most common presenting symptoms were decreased vision (15/16 patients) and pain (15/16 patients); all cases exhibited conjunctival injection, anterior chamber cell with hypopyon, and vitreous cell. Visual acuity at presentation ranged from 20/80 to hand motions, with 13 of 16 patients measuring worse than 20/100. The average time to presentation was 4.3 ± 3 days following injection (range 1–15 days) with no significant differences based on anti-VEGF agent or culture results, similar to other studies [5, 13]. All but one patient underwent primary vitreous tap with intravitreal injection of antibiotics (vancomycin and ceftazidime). The patient who underwent primary vitrectomy had the longest duration between injection and presentation (15 days) and visual acuity immediately prior to vitrectomy was counting fingers (CF). Of those who underwent primary tap/inject, 6 of the 15 total underwent subsequent pars plana vitrectomy, with the decision to proceed to surgery based on persistent vitritis.

**Table 1:** Summary of patients with endophthalmitis following intravitreal injection of anti-VEGF agent

Patient	Diagnosis	Medication	Pre-injection VA	VA at presentation	Days to presentation	Treatment	Culture results	Continued anti-VEGF?	Final VA	Length of follow-up (months)
1	BRVO/CME	Bevacizumab	20/50	CF	2	Tap/inject	CONS	N	20/40 − 1	12
2	BRVO/CME	Bevacizumab	20/100	HM	3	Tap/inject, then PPV	CONS	Y	20/125	23
3	CRVO/CME	Bevacizumab	20/40	CF	15	PPV	CONS	N	HM	26
4	DME	Bevacizumab	20/60	CF	4	Tap/inject	CONS	Y	20/80	22
5	NVAMD	Bevacizumab	20/40 − 2	20/80	3	Tap/inject	CONS	Y	20/80	25
6	Radiation retinopathy s/p plaque therapy for MM	Ranibizumab	20/200	HM	3	Tap/inject	CONS	Y	20/250	11
7	NVAMD	Ranibizumab	20/100	CF	7	Tap/inject, then PPV	CONS	N	20/160, ph 20/125	13
8	DME	Bevacizumab	? (elsewhere)	HM	4	Tap/inject, then PPV	CONS	Y	20/40	10
9	CRVO/CME	Ranibizumab	20/63	CF	3	Tap/inject	*Corynebacterium*	Y	20/80	23
10	MC/CNV	Ranibizumab	20/25	HM	1	Tap/inject, then PPV	*Strep viridans*, *Neisseria*	Y	20/160	16
11	NVAMD	Bevacizumab	? (elsewhere)	20/400	4	Tap/inject, then PPV	*E. coli*, *Enterobacter cloacae*	N	20/50 + 2	4
12	NVAMD	Bevacizumab	20/126	20/100	4	Tap/inject, then PPV	*Lactococcus garvieae*, CONS	Y	20/40	18
13	NVAMD	Bevacizumab	20/80	CF	5	Tap/inject	No growth	Y	20/150	22
14	CRVO/CME	Bevacizumab	20/250 − 3	HM	4	Tap/inject	No growth	N	HM	4
15	NVAMD	Ranibizumab	20/200 − 2	1/200	4	Tap/inject	No growth	N	CF	21
16	CRVO/CME	Ranibizumab	?	20/100	3	Tap/inject	No growth	Y	?	1

Summary of patients with endophthalmitis following intravitreal injection of anti-VEGF agent

*Abbreviations*: *VA *visual acuity, *BRVO* branch retinal vein occlusion, *CRVO* central retinal vein occlusion, *CME* cystoid macular edema, *DME* diabetic macular edema, *NVAMD* neovascular age-related macular degeneration, *MC* multifocal choroiditis, *CNV* choroidal neovascularization, *MM* malignant choroidal melanoma, *CF* counting fingers, *HM* hand motions, *PPV* pars plana vitrectomy, *CONS* coagulase-negative Staphylococcus

Vitreous or anterior chamber cultures were positive in 12 of the 16 cases (75 % total; 11 cases with positive vitreous culture, 1 case with positive aqueous tap). The most commonly isolated organism in our series was coagulase-negative *Staphylococcus* (8/16, or 50 % of all cases), consistent with previous literature [3, 4, 6]. Culture positivity did not correlate with poorer visual acuity at presentation or recovery, also consistent with prior literature. Of the remaining four culture-positive cases, several atypical organisms were identified. Patient #9 grew *Corynebacterium* in the enrichment broth only from anterior chamber aspirate and was managed with tap/inject alone. However, the remaining three cases associated with non-*Staphylococcus* bacteria required pars plana vitrectomy after initial tap/inject. *Strep viridans* has been noted to cause particularly fulminant intraocular infection, and unsurprisingly, the patient whose vitreous aspirate culture yielded *Strep viridans* and *Neisseria* (patient #10) exhibited an initial dramatic decrease in vision at presentation then persistent dense vitritis following tap/inject and a further decline in visual acuity to light perception, requiring vitrectomy 3 days later. Interestingly, the organisms isolated in the vitreous culture from this patient did demonstrate susceptibility to vancomycin despite lack of clinical improvement following initial injection of intravitreal vancomycin and ceftazidime. Patient #11 developed symptoms 1 day following the 21st intravitreal injection of bevacizumab for neovascular AMD and underwent tap/inject, both performed elsewhere, before presenting to our institution 4 weeks later and undergoing vitrectomy for non-clearing vitritis with vitreous cultures positive for *E. coli* and *Enterobacter cloacae*. Patient #12 presented 4 days after a second intravitreal injection of bevacizumab for neovascular AMD with concerns of only mild discomfort and redness and no change in vision compared with pre-injection acuity. Given the patient’s mild symptoms, treatment was initiated with topical steroids for 24 h, then tap/inject was performed when the patient developed a hypopyon the next day. Cultures from the vitreous aspirate grew *Lactococcus garvieae* and coagulase-negative *Staphylococcus*. Vitrectomy was performed 5 days after presentation in the setting of persistent dense vitritis. To our knowledge, *Enterobacter cloacae* and *Lactococcus garvieae* have not been described previously as causative agents in infectious endophthalmitis following intravitreal injection of an anti-VEGF agent. Both patients whose intraocular cultures grew these unusual organisms had persistent dense vitritis following tap/inject, leading to vitrectomy several days to weeks later. In spite of this, patient #11 and patient #12 ultimately achieved a best-corrected visual acuity of 20/50 + 2 and 20/40, respectively, at most recent follow-up.

**Fig. 2:**
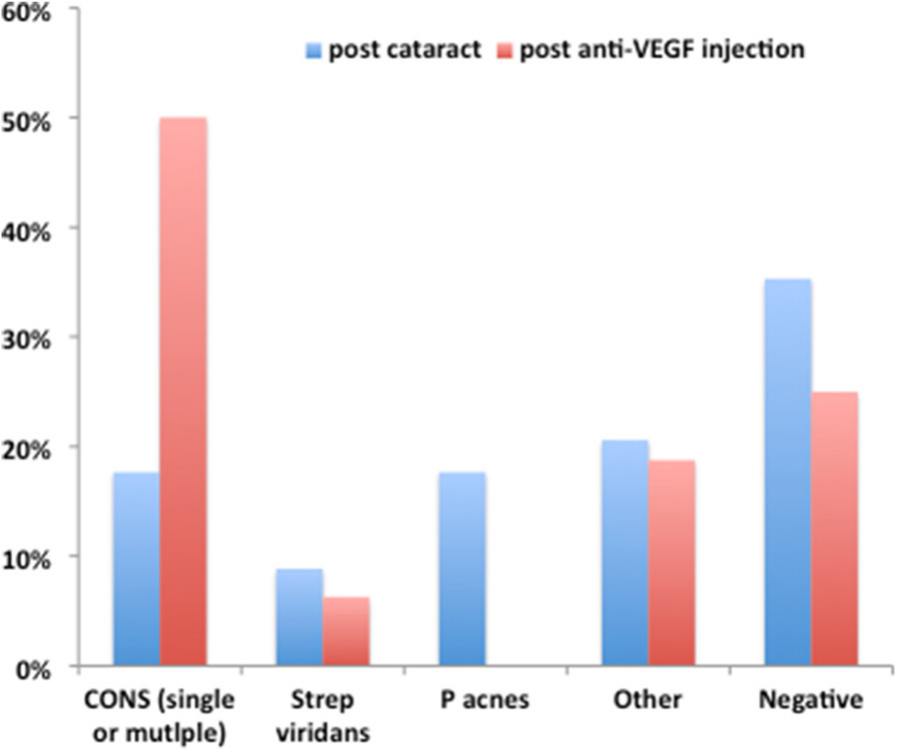
Comparison of microbes isolated from intraocular sampling between cases of endophthalmitis occurring post-injection versus following cataract surgery

Of all cases of endophthalmitis occurring after cataract surgery, 64.7 % (22/34) were culture-positive, with coagulase-negative *Staphylococcus* and *Propionibacterium acnes* accounting for the majority of positive culture results. Comparison with microbial culture results from endophthalmitis cases following anti-VEGF injections is shown in Fig. 2.

## Discussion

As VEGF antagonism has become the mainstay of treatment for highly prevalent retinal diseases characterized by neovascularization and retinal vascular leakage such as neovascular AMD, diabetic macular edema, and retinal vein occlusions, recent attention has focused on the associated risk of infectious endophthalmitis with intravitreal injection of these agents. Although several large retrospective analyses have indicated that the rate of endophthalmitis is low (approximately 0.05 % or 5 in 10, 000), for any individual patient potentially receiving repeated monthly injections, the statistical risk of iatrogenic infection increases. The use of pre- and post-procedure topical antibiotics for prophylaxis has been debated and recent evidence argues against its utility in improving visual outcomes [2, 17, 18]. Widespread use of prophylactic antibiotics may also contribute to the emergence of antibiotic-resistant organisms and higher rates of antibiotic treatment failure.

Here, in our 7-year retrospective analysis at the Wilmer Eye Institute at Johns Hopkins Hospital, we report that intravitreal injections of anti-VEGF agents comprised 8.5 % of all cases of endophthalmitis over the 7-year period during which anti-VEGF treatment has become frequently performed. A recent review of cases from another institution similarly found that anti-VEGF injections accounted for 11 % of cases of culture-positive endophthalmitis [19]. All but one patient in our series underwent initial treatment with tap/inject, and six of the remaining required subsequent vitrectomy.

Of the 75 % of cases of endophthalmitis following anti-VEGF injection which were culture-positive, two thirds (8/12) grew coagulase-negative *Staphylococcus* as the causative agent, consistent with prior reports. One case grew both *Strep viridans* and *Neisseria* supporting the recent suggestion that respiratory flora may comprise a significant potential source of infection. However, in the remainder of cases, we identified several unusual organisms. To our knowledge, this is the first report of intraocular *Corynebacterium*, *Enterobacter cloacae*, or *Lactococcus garvieae* infection following intravitreal anti-VEGF injections. *Corynebacterium* is part of normal conjunctival flora and has been isolated in cases of endophthalmitis following cataract surgery [20, 21]. *Enterobacter cloacae* is a gram-negative commensal organism which inhabits the human gastrointestinal tract and has been described previously in cases of endophthalmitis following cataract surgery and trauma [22–24]. *Lactococcus garvieae*, the most unexpected of our isolates, are gram-positive catalase-negative cocci which may be found in the human GI tract but typically act as disease-causing pathogens in fish. Human infection is rare but most commonly presents as infective endocarditis involving either native or prosthetic valves with lesser manifestations of intracranial hemorrhage, liver abscess, diverticulitis, peritonitis, and spondylodiscitis [25–27]. Transmission to humans may occur via ingestion of contaminated fish in conjunction with compromise of the normal immune barrier function of the host GI tract, leading to bacteremia and seeding of target organs. *Lactococcus garvieae* has not been isolated from intraocular cultures previously, and we report here the first identification of this organism as a pathogen in post-injection endophthalmitis. There were several atypical features of this patient’s presentation compared with endophthalmitis caused by other organisms, including the notable absence of pain and the minimal effect on visual acuity. This patient’s persistent vitritis following tap/inject also necessitated vitrectomy, but the visual outcome was excellent. Our data suggest that atypical organisms should be suspected in patients with dense non-clearing vitritis following tap/inject.

Comparison with organisms isolated in cases of endophthalmitis following cataract surgery did not yield any significant differences in our series, with coagulase-negative *Staphylococcus* comprising a large proportion in both contexts. Interestingly, we found that *P. acnes* comprised a large percentage of post-cataract endophthalmitis; this may be due to the inclusion of all cases of endophthalmitis occurring post-operatively, regardless of chronicity, as well as the tertiary care nature of our institution. Analyses of our endophthalmitis cases also suggest that a variety of pathogens may be the etiologic agents in post-injection endophthalmitis.

We conducted this comparison in an effort to assess the applicability of the EVS to the management of post-injection endophthalmitis. The findings of the EVS may have limited generalizability with regard to infectious endophthalmitis following intravitreal injection for several reasons. First, as has been long appreciated, the techniques of pars plana vitrectomy have improved significantly since the time of publication of the EVS, and clinicians today may be more apt to perform primary vitrectomy in a patient with endophthalmitis given the relative convenience and ease of the procedure with modern techniques. Second, the baseline visual potential of patients with post-VEGF endophthalmitis is often worse than that of those undergoing cataract surgery, for example, patients with subretinal fibrosis from neovascular AMD. We found that initial visual acuity did not predict success of recovery after tap/inject versus vitrectomy and that all patients did well when managed with primary tap/inject, though some required subsequent vitrectomy. Visual outcomes were stable enough that anti-VEGF injections were resumed in 10 of the 16 cases. Others have also suggested that in cases of post-injection endophthalmitis, the outcomes of primary tap/inject and vitrectomy are similar [28].

Our study has several limitations. First, these data rely upon retrospective review of billing codes to identify endophthalmitis cases and are dependent upon accuracy and consistency in physician coding. Second, the false negative rate of vitreous aspirate cultures may limit attempts to identify all potential causative micro-organisms associated with endophthalmitis. Finally, our study was not designed to directly compare outcomes of post-injection endophthalmitis patients treated with vitrectomy versus those treated with primary tap/inject—a large-scale prospective randomized trial would be needed to address this. However, the overall incidence of endophthalmitis following anti-VEGF injection is fortunately very low and such a prospective randomized clinical trial would likely not be feasible.

## Conclusions

Endophthalmitis following intravitreal injection of anti-VEGF agents represents an uncommon but serious complication of a procedure that has become routine in retinal practice. In the absence of current evidence-based guidelines, additional data regarding causative micro-organisms and clinical course may help inform management. Moreover, the emergence of more atypical pathogens has been suggested in the literature. Overall, our data suggest that post-injection endophthalmitis can be managed successfully with initial vitreous tap and injection of intravitreal antibiotics. Anti-VEGF therapy can be successfully resumed following endophthalmitis treatment. Unusual organisms should be suspected in cases of persistent vitritis following treatment, and these cases may require vitrectomy.
